# Interactions of *Aspergillus fumigatus* Conidia with Airway Epithelial Cells: A Critical Review

**DOI:** 10.3389/fmicb.2016.00472

**Published:** 2016-04-07

**Authors:** Carys A. Croft, Luka Culibrk, Margo M. Moore, Scott J. Tebbutt

**Affiliations:** ^1^Centre for Heart Lung Innovation, St. Paul’s Hospital, University of British Columbia, VancouverBC, Canada; ^2^Department of Biological Sciences, Simon Fraser University, BurnabyBC, Canada; ^3^Prevention of Organ Failure Centre of Excellence, VancouverBC, Canada; ^4^Department of Medicine, Division of Respiratory Medicine, University of British Columbia, VancouverBC, Canada

**Keywords:** *Aspergillus fumigatus*, conidia, airway epithelial cell, aspergillosis, virulence, immunity, host–pathogen interactions

## Abstract

*Aspergillus fumigatus* is an environmental filamentous fungus that also acts as an opportunistic pathogen able to cause a variety of symptoms, from an allergic response to a life-threatening disseminated fungal infection. The infectious agents are inhaled conidia whose first point of contact is most likely to be an airway epithelial cell (AEC). The interaction between epithelial cells and conidia is multifaceted and complex, and has implications for later steps in pathogenesis. Increasing evidence has demonstrated a key role for the airway epithelium in the response to respiratory pathogens, particularly at early stages of infection; therefore, elucidating the early stages of interaction of conidia with AECs is essential to understand the establishment of infection in cohorts of at-risk patients. Here, we present a comprehensive review of the early interactions between *A. fumigatus* and AECs, including bronchial and alveolar epithelial cells. We describe mechanisms of adhesion, internalization of conidia by AECs, the immune response of AECs, as well as the role of fungal virulence factors, and patterns of fungal gene expression characteristic of early infection. A clear understanding of the mechanisms involved in the early establishment of infection by *A. fumigatus* could point to novel targets for therapy and prophylaxis.

## Introduction

*Aspergillus fumigatus* is a saprotrophic filamentous fungus that plays an important environmental role in the carbon and nitrogen cycles through the decomposition of organic matter. Although *A. fumigatus* is not the most prevalent species of *Aspergillus*, it is one of the most ubiquitous, found in the soil of vastly different environments from the northern tundra to the tropics (Pringle et al., 2005). It is also able to grow in buildings, including hospitals (Araujo et al., 2010). A cycle of sexual reproduction has been characterized in this species (O’Gorman et al., 2009); nevertheless, the overall genetic variation between different isolates is comparatively low (Rydholm et al., 2006). *A. fumigatus* is primarily spread through the release of conidia; these are small, asexually produced haploid spores approximately 2–3 μm in diameter that can be disseminated by air currents (Mullins et al., 1976).

Species of the genus *Aspergillus* can cause aspergillosis in humans, a range of illnesses primarily affecting those with pre-existing conditions or compromised immune systems (Latgé, 1999). In addition to *A. fumigatus* a number of *Aspergillus* species are able to cause invasive aspergillosis including *A. niger*, *A. flavus*, *A. nidulans*, and *A. terreus*. However, *A. fumigatus* is implicated in up to 90% of all cases of aspergillosis (Perfect et al., 2001) suggesting that it has specific virulence factors enabling it to more efficiently colonize immunocompromised hosts. The disease process and symptoms depend very much upon the condition of the host (**Table 1**). Allergic Broncho Pulmonary Aspergillosis, or ABPA, is most common in patients with allergic asthma or cystic fibrosis, and manifests as a severe allergic reaction which can result in lung damage (Kumar, 2003). Fungal growth that remains localized within the lungs is defined as chronic pulmonary aspergillosis (CPA) and includes the growth of an aspergilloma, or fungal ball (Patterson and Strek, 2014). Though such conditions may be asymptomatic, should there be damage to the lung, life threatening hemoptysis may ensue which would necessitate surgery (Soubani and Chandrasekar, 2002). The most severe disease caused by *A. fumigatus* is invasive aspergillosis (IA) that involves the invasion of fungal hyphae into tissue and, in some cases, hematogenous spread to other organs, particularly the brain (Latgé, 1999). The primary site of infection is the lung. Infection of the skin and cornea may also occur, but fungal colonization of these sites is much less frequent. IA is rare in healthy individuals and almost exclusively affects patients with compromised immune systems. The greatest risk factors for developing IA are neutropenia, allogeneic hematopoietic stem cell transplant, or solid organ (in particular lung) transplant, hematological malignancy, and cytotoxic cancer chemotherapy. Patients with chronic granulomatous disease (CGD) and advanced AIDS also have an elevated risk of developing IA, as do patients receiving high-dose corticosteroid treatment (Kousha et al., 2011). Though mortality rates range from 30 to ≥90% depending upon the underlying condition of the patients, a paucity of effective treatments combined with the already poor state of patient health often results in a poor prognosis (Taccone et al., 2015). Other conditions caused by *Aspergillus* also exist with most having symptoms on a continuum between the conditions described above (Kosmidis and Denning, 2014).

**Table 1:** The primary manifestations of aspergillosis.

Disease Name	Risk factors	Symptoms/Manifestations	Reference
Allergic broncho pulmonary aspergillosis (ABPA)	• Allergic asthma	• Wheezing	Patterson and Strek, 2010
	• Cystic fibrosis	• Coughing	
		• Bronchiectasis	
		• Generalized allergic response	

Chronic pulmonary aspergillosis (CPA)	• Mycobacterial infection	• Asymptomatic	Patterson and Strek, 2014
	• COPD (Chronic obstructive pulmonary disease)	• Aspergilloma (fungal ball)	
	• Pre-existing lung cavity	• Aspergillus nodule	
	• Conditions impairing clearance	• Expanding, thick walled cavities	

Invasive aspergillosis (IA)	• Granulocytopenia	• Fever	Kousha et al., 2011
	• HIV/AIDS	• Dyspnea	
	• Immunosuppression	• Chest pain	
	• Cancer (particularly cancer of the blood)	• Disseminated fungal growth on multiple organs	


**FIGURE 1:**
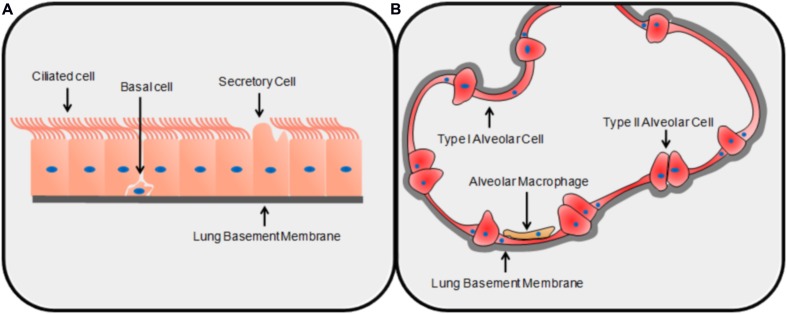
**The structures of the lung epithelium.** The basic structure and primary cell types of the bronchial **(A)** and alveolar **(B)** epithelia.

Conidia are the infectious particles of *A. fumigatus*. Their small size allows them to bypass mucociliary clearance and penetrate the lung alveoli (Latgé, 2001). It has been estimated that most people inhale up to several hundred conidia each day, and conidia have been isolated from the sputum of healthy and unaffected individuals (Mullins and Seaton, 1978; Latgé, 1999). The initial interaction between the conidium and the environment of the lung is important; in most healthy individuals, this interaction results in clearance whereas in susceptible patients, infection ensues (Fukahori et al., 2014). Due to the structure of the lung, the first cell encountered by a conidium is most likely to be a type of airway epithelial cell (AEC), either bronchial or alveolar. The bronchial epithelium is pseudostratified and made up of columnar epithelial cells of three main categories: ciliated cells, secretory cells, and basal cells (Knight and Holgate, 2003) (**Figure 1**). The alveoli are composed primarily of type I alveolar epithelial cells: thin, non-dividing squamous cells which cover ∼95% of the alveolar surface area despite being the least common of all major cell classes in the lungs (Crapo et al., 1982) (**Figure 1**). Type II alveolar cells are cuboid and secrete surfactant; they are the most common alveolar cell type and are the progenitors of type I alveolar epithelial cells (Crapo et al., 1982). The epithelial cell types that have been most studied in the context of pulmonary infection with *A. fumigatus* are bronchial epithelial cells and type II alveolar epithelial cells. Although alveolar macrophages patrol the alveoli and are demonstrably able to phagocytose and destroy conidia (Volling et al., 2011; Rammaert et al., 2015), because they constitute only ∼5% of total cell number in the alveoli, they are unlikely to be the first cell type encountered by the fungus (Crapo et al., 1982). Therefore, in this review paper, we have focused on the early interactions of conidia with AECs prior to significant hyphal growth. We cover areas related to conidial adhesion, internalization, the induction of an immune response, the roles of specific virulence factors, and patterns of gene expression that characterize this interaction.

## Adhesion

**FIGURE 2:**
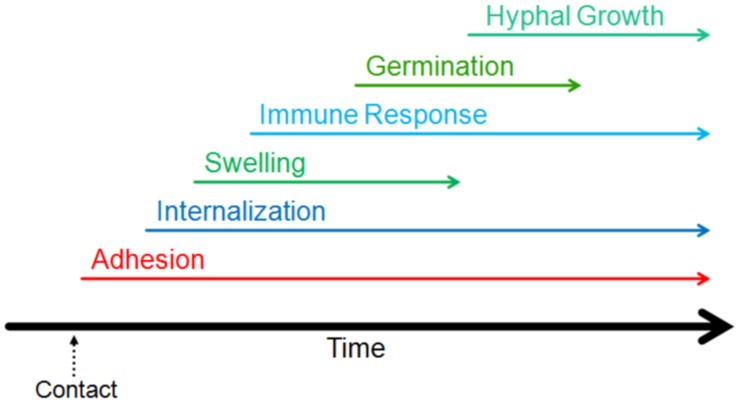
**Timeline of the events of the early interaction.** The initiation of different stages of interaction of *Aspergillus fumigatus* with host lung are indicated on a relative timeline. The events shown in the figure have been demonstrated to occur within the first 16 h of contact.

The first step in the process of infection involves adhesion to the host (**Figure 2**). Adhesion of the pathogen to the host, whether it be to the cell or the surrounding matrix of macromolecules, is important for the growth, persistence, and pathogenesis of fungal infections (de Groot et al., 2013). Conidia have been shown to adhere preferentially to proteins and carbohydrate moieties found on AECs as well as components of the basal lamina (DeHart et al., 1997; Wasylnka and Moore, 2000; Sheppard, 2011). Though it is typically not exposed in healthy lungs, the basal lamina is often exposed in those at risk of aspergillosis; this could provide another method of molecular adherence enabling fungal persistence and contributing to these individuals’ risks of aspergillosis (Bromley and Donaldson, 1996). Below, we have summarized a number of molecular mechanisms mediating conidial adherence that have been proposed or characterized.

Several proteins on the conidial surface have been demonstrated to mediate adherence to cellular components. One example is the extracellular/secreted Fucose-specific Lectin A, or FLeA, that is able to bind oligosaccharides containing fucose moieties much like its homolog AAL (*Aleuria aurantia* lectin; Houser et al., 2013). Fucose residues are present on host cell surface *N*-glycans, and thus provide a potential site of attachment for this lectin. Extracellular thaumatin domain protein AfCalAp (Afu3g09690) is another adhesin that has been implicated in conidial adhesion to laminin (Upadhyay et al., 2009). Anti-AfCalAp antibodies have been shown to bind conidia, primarily swollen conidia, but demonstrate poor specificity for hyphae (Upadhyay et al., 2009). It has a signal peptide sequence indicating it is a secreted fungal protein and contains multiple cysteine residues, allowing for the possibility of a binding interaction to other components of the conidial cell wall through strong covalent interactions (Upadhyay et al., 2009). Many thaumatin-like proteins expressed in plants are able to bind and hydrolyze β-glucans as a form of anti-fungal defense (Ho et al., 2007). The thaumatin domain of AfCalAp could promote binding to β-glucans in the fungal cell wall (Upadhyay et al., 2009). The expression of AfCalAp during early stages of germination and its presence on the cell wall indicate that it may play a role throughout the early stages of interaction in the lung by binding to laminin. The conidial cell wall also contains a large number of carbohydrate moieties, some of which have been demonstrated to have a role in adherence. Sialic acids have been detected on the surface of various fungal pathogens, including *A. fumigatus* (Wasylnka et al., 2001). Sialic acids mediated adherence to components of the extracellular matrix, e.g., conidial adherence to fibronectin was drastically decreased upon sialidase treatment of spores, though this treatment increased adherence of conidia to the type II alveolar epithelial cell line (A549 cells; Warwas et al., 2007). Conidia bound to the glycosaminoglycan (GAG) binding domain of fibronectin, a cluster of amino acids able to bind negatively charged sugars at low ionic strength (Wasylnka and Moore, 2000). In keeping with this mechanism, negatively charged sugars inhibited the adherence of conidia to both fibronectin and the basal lamina (Wasylnka and Moore, 2000). Sialic acids may also prevent conidial adherence to components of the ECM through a pH effect inhibiting other methods of adhesion (Tiralongo et al., 2009). Further characterization of adherence mechanisms mediated by conidial carbohydrates is necessary to fully understand their role with regards to adhesion to components of the ECM and basal lamina.

Recent studies have identified the exopolysaccharide galactosaminogalactan, a component of the hyphal cell wall that is also secreted by the fungus, as essential for fungal adherence and virulence (Gravelat et al., 2013; Lee et al., 2014). Mutants deficient in enzymes that mediate synthesis of this heteropolysaccharide demonstrate a significant decrease in adherence to epithelial cells and decreased virulence, highlighting its importance as a fungal adhesin during both early- and late-stage hyphal growth (Gravelat et al., 2013). Loss of galactosaminogalactan was also demonstrated to increase the inflammatory response and decrease the *A. fumigatus* induced epithelial cell damage, possibly due to an increase in β-glucan exposure resulting from the loss of galactosaminogalactan (Gravelat et al., 2013). A novel glycoside hydrolase, Sph3, has been recently shown to be part of the co-regulated cluster of five genes that are responsible for the biosynthesis of galactosaminogalactan (Bamford et al., 2015). Further study is necessary to fully understand the impact of this exopolysaccharide on virulence during the first stages of infection.

*H*-ficolin is a soluble lectin-like opsonin involved in innate immunity that contains collagen-like and fibrinogen-like domains. *H*-ficolin is secreted by type II alveolar epithelial cells and has been found in human bronchiolar lavage fluid (Akaiwa et al., 1999). *H*-ficolin was able to bind *A. fumigatus* conidia in a calcium-dependant manner and best in acidic conditions; this interaction was substantially inhibited by L-fucose, D-mannose, and *N*-acetylglucosamine (Bidula et al., 2015). Conidia with adherent *H*-ficolin also adhered to a greater extent to alveolar type II A549 cells (Bidula et al., 2015). Finally, *E*-cadherin, a glycoprotein that mediates cell–cell adhesion in human epithelial cells, is also proposed to be involved in the adhesion of *A. fumigatus.* Knockdown of *E*-cadherin expression in A549 cells decreased the number of conidia bound to the cells (Xu et al., 2012; Yan et al., 2015). The fungal ligands are proposed to be two uncharacterized proteins of *A. fumigatus* (Yan et al., 2015).

A number of other conidial adhesins have been partially described. One example is a sialic acid-specific lectin purified from conidia (Tronchin et al., 2002). Other examples include proteins able to bind to basal lamina and extracellular matrix components such as laminin and fibronectin (Gil et al., 1996; Peñalver et al., 1996). Moreover, the *A. fumigatus* genome contains a number of putative adhesins (Gravelat et al., 2010; Chaudhuri et al., 2011). The role of these adhesins and their importance in the observed adherence of conidia to AECs has yet to be validated or described, and is an area that requires further study. It is also important to note that other fungal gene products have been demonstrated to be important in adherence, not as adhesins *per se*, but because they are involved in the synthesis and structure of the cell wall, e.g., enzymes involved in melanin synthesis (Amin et al., 2014) and the cell surface protein CspA, which regulates the structure of the conidial cell wall (Levdansky et al., 2010). Adherence of conidia to collagen and albumin is decreased upon loss of the conidial hydrophobin rodA, the component of the conidial cell wall that causes its hydrophobicity and helps mediate immune evasion (Thau et al., 1994). It is possible that rodA is able to mediate non-specific hydrophobic interactions with collagen and laminin, or that the loss of adherence is due to the altered cell wall morphology of the *ΔrodA* mutant (Thau et al., 1994). It is likely that there are a number of different ligands that mediate conidial adhesion, and further research into the structure and proteome of the conidial cell wall is needed to fully understand their relative importance in binding to host molecules.

## Internalization

**FIGURE 3:**
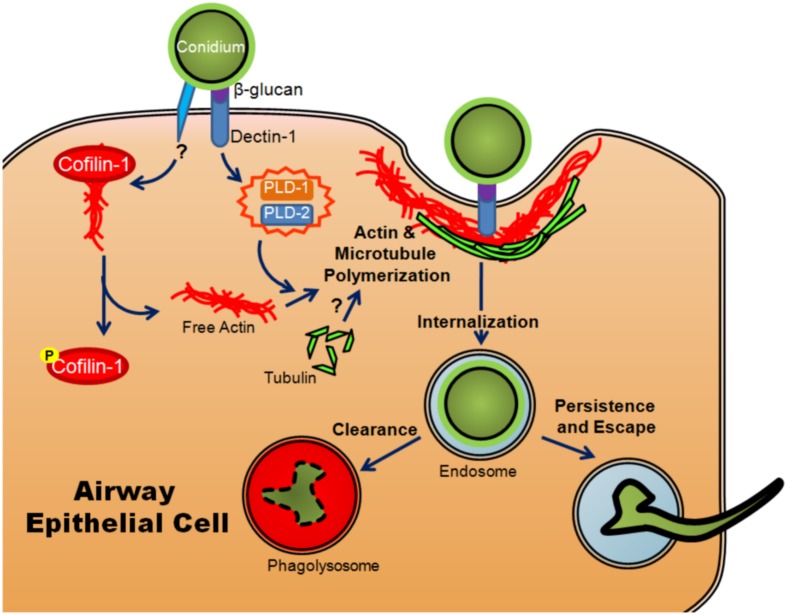
**Model of internalization of *A. fumigatus* conidia by airway epithelial cells.** Once conidia swell, β-glucan in the conidial cell wall is recognized by dectin-1, activating PLD-1 and PLD-2 (isoforms of phospholipase D) and promoting actin polymerization. Another, as yet uncharacterized signal results in the phosphorylation of cofilin-1, causing it to release actin. Conidia are then internalized through actin and microtubule polymerization and phagocytosis. Upon internalization, they are trafficked through the endosomal system where they are degraded and cleared from the host or alternatively, small numbers may persist, germinate and escape.

The role of intracellular uptake in fungal pathogenesis is poorly understood, particularly in comparison to viral and bacterial pathogens. It remains an area of interest because internalization is known to be a method of escape from the immune system used by many pathogens (Abel et al., 2011). It has been observed that conidia of *A. fumigatus* are taken up *in vitro* by professional phagocytes as well as typically non-phagocytic cells, including AECs (Paris et al., 1997; Wasylnka and Moore, 2002; Gomez et al., 2010). Cultured AECs have been shown to take up ∼30% of bound conidia (Wasylnka and Moore, 2002; Gomez et al., 2010). This internalization requires the use of both microtubules and actin polymerization, indicating a mechanism similar to phagocytosis (Wasylnka and Moore, 2003; Botterel et al., 2008) (**Figure 3**). Once they have been internalized, conidia are trafficked through the endosomal system of the cell to the phagolysosome (Wasylnka and Moore, 2003; Botterel et al., 2008) (**Figure 3**). Interestingly, although the majority of conidia are killed within the A549 cell, a small percentage survive and are able to germinate within the phagolysosome and ultimately re-enter the extracellular space (Wasylnka and Moore, 2003) (**Figure 3**).

The human isoforms of phospholipase D (PLD) have also been shown to participate in the internalization of conidia in alveolar type II cells. Upon exposure to conidia or β-glucan, PLD activity increased (Han et al., 2011). The increase was dependent on the presence of Dectin-1, a lectin-like pattern recognition receptor important in anti-fungal immunity (Han et al., 2011). The actin-binding protein, cofilin-1, has also been shown to be involved in internalization. Non-phosphorylated cofilin actively binds to actin and prevents its polymerization. Upon exposure of A549 cells to *A. fumigatus* conidia, levels of inactive phosphorylated cofilin increased while the total amount of cofilin-1 remained unchanged (Bao et al., 2015). Unlike PLD, the change in the ratio of phosphorylated cofilin-1 to unphosphorylated cofilin-1 was not mediated by exposure to β-glucan, implying that the inactivation of cofilin was mediated through a different signaling event (Bao et al., 2015). The involvement of both molecules appears to be essential for efficient internalization as silencing of the expression of either PLD or cofilin-1 resulted in a decreased conidial uptake (Han et al., 2011; Jia et al., 2014; Bao et al., 2015) (**Figure 3**). Another set of studies has shown that silencing of *E*-cadherin also decreased the phagocytosis of conidia by A549 cells (Xu et al., 2012; Yan et al., 2015). It is unclear whether or not *E*-cadherin directly mediated the internalization of conidia or merely increased the probability that conidia interacted with another receptor on the cell surface.

The importance of β-glucan and actin polymerization to the Dectin-1 mediated internalization of conidia was clearly demonstrated through the creation of a mutant lacking the pH responsive transcription factor PacC. Δ*pacC* conidia had an atypical distribution of β-glucan and were also internalized in much smaller proportions than their wild-type counterparts despite no observable change in adherence (Bertuzzi et al., 2014). This difference in internalization between the wild type and mutant correlated with differences in contact-dependant damage to the A549 cells; the Δ*pacC* conidia induced much less damage to co-cultured cells than the wild type conidia (Bertuzzi et al., 2014). Furthermore, inhibitors of actin polymerization and an anti-Dectin-1 antibody decreased both the amount of conidia internalized and the monolayer decay (Bertuzzi et al., 2014). *In vivo* evidence showed that a decrease of Dectin-1 expression in mice correlated with increased epithelial damage, suggesting that Dectin-1 has an important protective role despite the observed damage that arose from Dectin-1 dependent internalization of conidia *in vitro* (Rand et al., 2010; Sun et al., 2012; Carrion et al., 2013; Bertuzzi et al., 2014).

Transcriptomic studies on AECs have provided support for a role of the cytoskeleton in conidial internalization. An increase in the expression of genes involved in cytoskeleton rearrangement has been reported, including Activity Regulated Cytoskeleton-associated protein (ARC), early growth response protein 1 (EGR1; Chen et al., 2015), and α-actinin-2 (ACTN-2; Gomez et al., 2010). In addition, the effects on internalization of two of these genes, ARC and EGR1, were tested using siRNA (Chen et al., 2015). Internalization in cells with silenced ARC was reduced by 20% and by 40% in cells with silenced EGR1 (Chen et al., 2015).

There is clear and consistent evidence of internalization of conidia by many types of AECs including cells grown in an air–liquid interface, growth conditions that promote cell differentiation and pseudostratification (Gruenert et al., 1995). These studies also reported comparable rates of internalization to previously performed studies on monolayer cultures (Botterel et al., 2008; Khoufache et al., 2010). Despite the consistent *in vitro* evidence of internalization of conidia by AECs, there has been no clear observation of *in vivo* internalization of conidia by AECs, whereas macrophages have been shown to clearly phagocytose conidia both *in vitro* and *in vivo* (Hasenberg et al., 2011; Rammaert et al., 2015). A study examining an organ culture model did find some internalization in this *in vitro* setting (Amitani and Kawanami, 2009). However, a recent study using transmission electron microscopy (TEM) that examined the early *in vivo* interactions between the bronchial epithelium in mice and *A. fumigatus* reported a lack of internalization of conidia (Rammaert et al., 2015). This lends credence to the possibility that internalization may not be relevant to infections *in vivo*. The complex cellular environment *in vivo* may cause AECs to behave differently upon exposure to conidia. Furthermore, type I cells, the epithelial cell most likely to be encountered by conidia *in vivo* may not take up conidia at the same rate as type II cells. It is also possible that internalization does occur *in vivo*, but at a much lower frequency than is observed *in vitro* and TEM may not be sufficiently sensitive to detect lower rates of internalization. Different cell types may also internalize particles to different extents. For example, Rammaert et al. (2015) examined the bronchial epithelium but not the alveolar epithelium. The alveolar epithelium has been shown to take up both inert particles and bacteria *in vivo* (Churg, 1996; Hall et al., 2007). In addition, *Francisella tularensis*, a Gram-positive bacterium that can act as a facultative intracellular pathogen, was internalized by the alveolar epithelium but not the bronchial epithelium (Hall et al., 2007). The inherent differences between bacteria and fungi may influence their relative uptake by various cell types; nevertheless, the evidence that alveolar epithelial cells internalize bacteria *in vivo* and the consistent evidence that fungal conidia are taken up by non-phagocytic cells *in vitro* support the need for more research into conidial internalization by AECs *in vivo*.

## Airway Epithelial Cell Immune Responses

Though it was previously thought to function mainly as an anatomical barrier to invasion by pathogens, the respiratory epithelium has been demonstrated to participate in the immune response (Vareille et al., 2011; Heyl et al., 2014). AECs are able to express chemokines, cytokines, and proteins involved in innate immunity. In addition, they express a number of different receptors able to recognize pathogens. Consequently, AECs are able to affect and induce a specific type of response from components of the adaptive and innate immune systems (Schleimer et al., 2007). AECs are likely important players in the early immune response to *A. fumigatus* infection. In addition, other types of epithelial cell responses to both the conidia and immune mediators have been demonstrated through transcriptome studies. Genes and pathways involved in the prevention of oxidative damage and repair, including MGST1 (Microsomal Glutathione *S*-Transferase 1), were up-regulated in human AECs upon exposure to conidia (Gomez et al., 2010; Oosthuizen et al., 2011). Pathways involved in cell cycle progression and mitosis were down-regulated upon exposure to conidia (Gomez et al., 2010; Oosthuizen et al., 2011). These patterns are consistent with the response to a pathogen, i.e., up-regulation of the immune response and down-regulation of genes involved in the cell cycle and mitosis (Gomez et al., 2010; Oosthuizen et al., 2011; Fekkar et al., 2012; Chen et al., 2015). Pathway-specific genes are reviewed in more detail below.

### Pattern Recognition Receptors

Pattern recognition receptors, or PRRs, are essential for the detection of pathogens through the recognition of PAMPs, pathogen associated molecular patterns. Different PRRs activate different pathways and responses specific to certain types of pathogens, and are therefore essential in the detection and clearance of pathogens. PRR activation can initiate the expression of many different proteins and affect a number of different pathways; however, their primary effect is to increase the expression of immune effectors such as cytokines (Mogensen, 2009). The specific cytokines released have additional downstream effects on the innate and adaptive immune responses, and thus PRR activation may lead to pathogen clearance or allow for the development of disease.

Dectin-1, in addition to its role in internalization as described above, is a PRR expressed on AECs (Sun et al., 2012; Heyl et al., 2014). Upon exposure of human bronchial cells to *A. fumigatus* conidia, an increase in Dectin-1 was observed (Sun et al., 2012). Concomitantly, up-regulation of genes encoding the pro-inflammatory cytokines IL-8 and TNFα as well as up-regulation of the beta defensin genes hBD-2 and hBD-9 was observed (Sun et al., 2012). Silencing of Dectin-1 corresponded with a decrease in the expression of these mediators (Sun et al., 2012). Although *A. fumigatus* conidia damaged type II alveolar epithelial cells in a Dectin-1 dependent manner, loss of this PRR is also correlated with increased lung damage and cell death *in vivo*, implying that Dectin-1 has a protective role (Bertuzzi et al., 2014). As Dectin-1 is also the major β-glucan receptor on macrophages (Brown et al., 2002), it is possible that the increased mortality in the Dectin-1^-/-^ knockout mice was related to the loss of Dectin-1 on phagocytes despite the use of corticosteroids to inhibit macrophage activity.

Long pentraxin 3 (Ptx3) is a soluble pattern recognition receptor that has an important role in the recognition, uptake, and killing of conidia by macrophages (Garlanda et al., 2002). Conidia with bound Ptx3 were phagocytosed by alveolar macrophages at a higher rate demonstrating that Ptx3 functions as an opsonin (Garlanda et al., 2002). Ptx3 also activates complement through the classical pathway by binding to C1q (Garlanda et al., 2002; Moalli et al., 2010). Ptx3 secretion can be elicited in both primary and cultured AECs by treatment with TNF-α, and in A549 cells upon exposure to conidia (Han et al., 2005; Chen et al., 2015). Due to its importance in the innate immune response to conidia, Ptx3 production by airway epithelia could play a key role in preventing fungal germination and subsequent invasion.

Toll-like receptors, or TLRs, are a family of conserved PRRs that are essential components of the innate immune system. Although they are predominantly expressed on leukocytes such as macrophages and dendritic cells, their expression in primary AECs has been detected (Ritter et al., 2005). The roles of several TLRs have been examined in the context of *A. fumigatus* exposure. TLR-2 has been shown to have an essential role in the expression of Dectin-1 on epithelial cells in response to *A. fumigatus*; silencing of TLR-2 resulted in a decrease in Dectin-1 expression (Sun et al., 2012). Roles for TLR-2 have also been demonstrated in the response of corneal epithelial cells to *A. fumigatus* as well as in macrophages and other cell types (Chai et al., 2011; Wu et al., 2014). TLR-3 is a toll-like receptor that recognizes double-stranded RNA and is primarily involved in the recognition of viruses. TLR-3 has also been implicated in the recognition of conidia. Induction of IFNβ and IP-10, cytokines associated with an antiviral response, increased in human bronchial epithelial cells upon exposure to conidia and internalization of conidia; a similar induction was observed upon stimulation with a TLR-3 agonist (Beisswenger et al., 2012). The TLR-3/TRIF (TIR-domain-containing adaptor-inducing interferon-β) pathway in AECs has been demonstrated as particularly important in the development of a protective and effective response to *A. fumigatus*. Loss of either TRIF or TLR-3 on non-hematopoietic cells resulted in a much more persistent inflammatory response, a greater susceptibility to fungal allergy and a greater fungal burden when compared to wild type or mice deficient in MyD88, another TLR adaptor protein (de Luca et al., 2010). It has been shown that TRIF-deficient epithelial cells and mice were unable to activate IDO (indoleamine 2,3-dioxygenase; de Luca et al., 2010), a rate limiting step of tryptophan catabolism through the kynurenine pathway, through which dendritic cells reduce excessive adaptive inflammatory responses (Montagnoli et al., 2006). Both a TLR-3 agonist and swollen conidia induced IDO protein expression *in vitro* through the NF-κB pathway (de Luca et al., 2010). Elucidating the roles of TLRs on AECs in the response to *A. fumigatus* will further clarify the role of AECs in the immune response.

### Secreted Immune Effectors

Among the gene ontology terms shown to be up-regulated in transcriptomic studies of the AEC response to conidia, the immune response appears consistently, due in part to the up-regulation of pro-inflammatory cytokines and chemokines (Gomez et al., 2010; Oosthuizen et al., 2011; Chen et al., 2015). The importance of a number of these cytokines in a protective host response and relevance to the disease progression of aspergillosis has been confirmed experimentally. For example, the inability to produce CC motif chemokine 3 (CCL3) is associated with increased fungal burden in the lungs (Shahan et al., 1998). Increased expression of IL-6 has also been reported in AECs exposed to conidia (Gomez et al., 2010; Oosthuizen et al., 2011; Chen et al., 2015). Moreover, IL-6 deficiency is associated with increased susceptibility to aspergillosis (Cenci et al., 2001). Transcriptomic studies have also demonstrated an increase in expression of other cytokines and chemokines; however, the mechanism of their induction has not yet been elucidated (Gomez et al., 2010; Oosthuizen et al., 2011; Chen et al., 2015). In general, the up-regulated cytokines and chemokines are involved in the recruitment of immune effector cells, such as macrophages and neutrophils, as part of the response to fungal infection (Gomez et al., 2010; Oosthuizen et al., 2011; Sun et al., 2012; Chen et al., 2015).

While the mechanisms resulting in the expression and secretion of numerous cytokines and chemokines in response to conidia are generally poorly characterized, the induction of interleukin-8 (IL-8) in AECs has been described in relative detail. IL-8, or CXCL8, is a pro-inflammatory chemokine that acts as a neutrophil chemoattractant and has been studied in the context of fungal infections (Ali et al., 2006). A significant increase in IL-8 expression in response to *A. fumigatus* conidia has been demonstrated in both AECs (Balloy et al., 2008; Sharon et al., 2011; Sun et al., 2012) and corneal epithelial cells (Peng et al., 2015). Dectin-1 likely plays a key role in the induction of IL-8 because expression of IL-8 peaks after ∼8 h of exposure to *A. fumigatus* conidia (Balloy et al., 2008), the time at which most conidia are swollen and thus have significantly more exposed β-glucan, a ligand for Dectin-1 (Hohl et al., 2005). In addition, it has been shown that the opsonisation of conidia by *H*-ficolin also increased the amount of IL-8 expressed by A549 cells (Bidula et al., 2015). Control of IL-8 synthesis is regulated through several pathways, primarily the pI3K, p38 MAPK, and ERK1/2 pathways (Balloy et al., 2008; Bidula et al., 2015). IL-8 expression in response to *A. fumigatus* is under the control of the transcription factors NF-κB and AP-1 (Balloy et al., 2008). The demonstrable expression of IL-8 in the lungs of mice infected with *A. fumigatus* strongly suggests that it is an essential part of the response leading to fungal clearance (Mehrad et al., 2002).

The up-regulation and increased secretion of antimicrobial peptides and other effectors of the innate immune system has been observed in the response to *A. fumigatus*. Antimicrobial peptides have many and varied functions, from direct antimicrobial activity to modulation of the immune response (Steinstraesser et al., 2011; Nakatsuji and Gallo, 2012). AECs showed increased expression of the human beta defensins hBD-2 and hBD-9 upon exposure to conidia (Alekseeva et al., 2009; Sun et al., 2012). The expression of these defensins increased as conidia swelled, thereby exposing more immuno-reactive surface components (Alekseeva et al., 2009). Another study that examined the secretome of bronchial epithelial cells found an increase in cathepsin B and cathepsin D release upon exposure to conidia (Fekkar et al., 2012). The up-regulation of two genes encoding matrix metallopeptidases, MMP1 and MMP2, has also been demonstrated in AECs upon exposure to conidia (Gomez et al., 2010). Matrix metallopeptidases play a key role in tissue remodeling after damage and protein processing (Herrera et al., 2013); the differential expression of MMPs in response to a number of bacterial pathogens has also been demonstrated, suggesting that these enzymes play an important general role in the host response to pathogens (Vanlaere and Libert, 2009).

### The Protective Response

While the recruitment of immune cells and expression of pro-inflammatory cytokines is essential for a protective immune response to conidia, an inflammatory response is also associated with tissue damage and can actually aid in fungal persistence (Bromley and Donaldson, 1996; Fukahori et al., 2014). Over-expression of pro-inflammatory cytokines, including some induced upon exposure to *A. fumigatus*, is also characteristic of asthmatic individuals and those with cystic fibrosis who are at risk of developing ABPA (Neveu et al., 2011; Rincon and Irvin, 2012). For example, a T helper 2 cell bias has been demonstrated in a mouse model of cystic fibrosis upon exposure to *A. fumigatus* (Chaudhary et al., 2012). In the same study, AECs with a non-functional cystic fibrosis transmembrane conductance regulator (CFTR) showed increased inflammatory cytokine expression but a reduced ability to kill internalized conidia (Chaudhary et al., 2012). As noted above, the importance of the protective TLR-3/TRIF pathway has also been demonstrated in a mouse model (de Luca et al., 2010). These authors also showed that a lack of this pathway was associated with a greater inflammatory Th2/Th17 adaptive response as opposed to a protective one mediated by T helper 1/T regulatory cells (de Luca et al., 2010).

Vitamin D has been demonstrated to modulate the immune response to *A. fumigatus.* Vitamin D has protective effects against respiratory pathogens upon its conversion to the active hormonal form, 1,25D_3_ (Kearns et al., 2015). The extent of conversion to active hormone increased in bronchial epithelial cells upon exposure to swollen conidia (Li et al., 2015). Furthermore, when compared to controls, addition of vitamin D at the same time as conidia increased the expression of the antimicrobial peptides cathelicidin (LL-37) and hBD-2 by these cells, while reducing the conidia induced expression of pro-inflammatory cytokines such as TNFα, IL-8, IL-6, and IL1B (Li et al., 2015). Thus, vitamin D altered the overall response of bronchial epithelial cells to reduce the potential for tissue damage and increase the expression of peptides with direct anti-microbial activity. This protective and anti-inflammatory effect of vitamin D has also been demonstrated *in vivo*; mice deficient in vitamin D sustained greater lung damage and had a higher rate of mortality when challenged with *A. fumigatus* conidia (Li et al., 2014).

## Aspergillus Fumigatus Virulence Factors and Epithelial Cells

The elucidation of *A. fumigatus* virulence has been challenging for a number of reasons. First, there is significant redundancy in a number of pathways. As a result, the fungus is able to compensate for the loss of a specific pathway and demonstrates little to no reduction in virulence, and knockout or knockdown of large gene families can be technically challenging. Second, a large portion of the fungal genome contains uncharacterized elements, e.g., many proteins are unannotated open reading frames (ORFs) or have a functional prediction based on sequence similarity to known proteins. Third, a number of genes which likely contribute to virulence are also essential for growth, making it difficult to generate loss-of-function mutants to study the direct effects. These latter gene products are not strictly considered to be virulence factors (Casadevall, 2005); however, they may contribute to growth only under specific nutrient conditions. Nevertheless, several pathways involved in the virulence and early establishment of infection have been described (Moore, 2013; Amich and Calera, 2014; Haas, 2014; Krishnan and Askew, 2014). The following sections will focus on virulence factors with regard to their impact on epithelial cell structure or function.

### Iron Uptake

Iron plays a key role in a number of essential cellular redox reactions in both the host cell and the pathogen; therefore, the ability to sequester iron from the host provides an important competitive advantage for a pathogen (Parrow et al., 2013). The healthy lung lumen has very little free iron; it is within the airway cells or bound to lactoferrin and transferrin in surfactant fluid. Even within the host cells, conidia that are trafficked through the endosomal pathways of AECs or professional phagocytes are in an environment with growth-limiting concentrations of iron (Zarember et al., 2007). *A fumigatus* genes involved in iron uptake were up-regulated when conidia were incubated with AECs (Oosthuizen et al., 2011). *A. fumigatus* is able to obtain iron in two ways: reductive iron assimilation and siderophore biosynthesis (Moore, 2013). Of the two, siderophore biosynthesis has been shown to be essential for virulence (Schrettl et al., 2004; Hissen et al., 2005), although mutants with only the ability to perform reductive iron assimilation are able to grow in media that contain high concentrations of iron (Schrettl et al., 2007). A conidial-specific siderophore, hydroxyferricrocin (HFC), involved in intracellular iron storage has been identified through mutation of genes in the siderophore biosynthetic pathway (Schrettl et al., 2007). The gene for HFC synthesis has also been demonstrated to be essential for fungal growth, highlighting the importance of iron metabolism in the growth and pathogenesis of *A. fumigatus* (Schrettl et al., 2007). The ability to take up and store iron in a competitive manner is essential to the ability of conidia to survive *in vivo*; it is not clear at present whether these genes are also essential for survival in the intracellular environment.

### Melanin

1,8-Dihydroxynaphthalene-melanin (DHN-melanin) is the secondary metabolite that gives conidia their characteristic gray–green color; it is also a virulence factor of *A. fumigatus* (Heinekamp et al., 2012). Melanized conidia are taken up in greater numbers by the type II alveolar epithelial A549 cell line when compared to non-melanized conidia (Amin et al., 2014). Interestingly, this is in direct contrast to the interaction of conidia with phagocytes, which take up non-melanized conidia in far greater numbers than the wild-type pigmented strains (Thywißen et al., 2011; Volling et al., 2011). Upon internalization, a small proportion of wild type conidia have been shown to survive in the acidic organelles of A549 pneumocytes (Wasylnka and Moore, 2003). Another study conducted by Amin et al. (2014) found only pigmentless conidia in the acidic organelles of the same cell type. It is interesting to note that the results of Amin et al. (2014) were obtained using a uracil auxotrophic strain of *A. fumigatus;* this strain cannot germinate within the endosomal system. Use of this strain prevented germination of the fungus, allowing for longer incubation periods; however, the inability to germinate has the potential to change the interaction of conidia with epithelial cells. Germination is known to elicit different responses than resting conidia due to the breakdown of the rodlet layer and exposure of immunogenic components of the cell wall (Alekseeva et al., 2009; Han et al., 2011; Sun et al., 2012; Li et al., 2015).

Prevention of host cell apoptosis has been observed with both bacterial and fungal pathogens (Faherty and Maurelli, 2008; Shlezinger et al., 2011). Exposure to live *A. fumigatus* conidia prevented induction of apoptosis in both alveolar and bronchial epithelial cell lines (Berkova et al., 2006; Féménia et al., 2009; Amin et al., 2014). In the context of aspergillosis, this anti-apoptotic effect could provide an advantage to the fungus by promoting its intracellular survival and persistence thereby allowing it to escape immune detection. Exposure to conidia promoted the degradation of the active 17 kDa caspase-3 fragment, and thus prevented TNF-α induced apoptosis (Berkova et al., 2006; Amin et al., 2014). Similarly, conidia prevented apoptosis in cells exposed to staurosporine in a caspase-3 independent manner (Berkova et al., 2006). The anti-apoptotic effect has been attributed to a soluble factor associated with or produced by conidia, as the supernatant from a conidial suspension also had anti-apoptotic effects on AECs (Féménia et al., 2009). Another study of the anti-apoptotic ability of *A. fumigatus* conidia on AECs showed that DHN-melanin was required (Amin et al., 2014). Melanized conidia have been previously shown to be anti-apoptotic in phagocytes (Volling et al., 2011). It is possible that the soluble anti-apoptotic factor, found by Féménia et al. (2009), was melanin that had been solubilized and released during the production of the supernatant. Further work is needed to confirm whether or not there are molecules in addition to DHN-melanin that act to prevent apoptosis of epithelia exposed to *A. fumigatus* conidia.

### Other Secondary Metabolites

Like many other fungi, *A. fumigatus* produces a number of different toxic secondary metabolites (Frisvad et al., 2009). Gliotoxin is commonly isolated from the tissues of patients with IA, but is primarily produced in hyphae (Geissler et al., 2013). Several toxins have been identified that are associated with conidia, some of which have toxic effects on mammalian cells. For example, trypacidin is a spore-borne toxin that causes necrosis of cultured A549 pneumocytes and human bronchial cells; effects of pure toxin were observed with an IC_50_ of 7 μM (Gauthier et al., 2012). Cell death was not mediated by apoptosis (Gauthier et al., 2012), an observation consistent with the observed anti-apoptotic effect of conidia (Berkova et al., 2006; Féménia et al., 2009; Amin et al., 2014). In addition, intermediates in the pathway of trypacidin synthesis were also shown to damage cultured AECs, albeit at higher concentrations (Gauthier et al., 2012). Verruculogen is another mycotoxin found associated with conidia (Khoufache et al., 2007; Gauthier et al., 2012). While its toxicity was significantly lower than that of trypacidin (Gauthier et al., 2012), it has been shown to disrupt the transepithelial resistance of polarized human nasal epithelial cells *in vitro*, though the significance of this effect in pathogenesis is presently unknown (Khoufache et al., 2007). In addition to the aforementioned compounds, when grown under environmentally relevant conditions, *A. fumigatus* conidia contained the ergot alkaloids fumigaclavine C, festuclavine, fumigaclavine A, and fumigaclavine B (in order of abundance) in amounts of >1% total conidial mass (Panaccione and Coyle, 2005). Interestingly, it has also been shown that fumigaclavine C induced apoptosis in cultured human breast cancer cells (MCF-7), an epithelial cell line, but this effect occurred at concentrations of 20 μM and above (Li et al., 2013). Although significant physiological effects have been demonstrated, the relevance of conidial toxins to *A. fumigatus* pathogenesis *in vivo* has yet to be elucidated.

### Extracellular Enzymes

Proteases and other enzymes able to cause damage to AECs and the environment of the lung could contribute to the expression of pro-inflammatory cytokines by AECs, or alter the activity of host defense proteins. The up-regulation of secreted proteins is significant throughout early growth (Bertuzzi et al., 2014). The majority of proteases are secreted during hyphal growth, and consistent with this, culture filtrate and hyphal cultures induced the greatest protease-mediated epithelial damage (Sharon et al., 2011; Bertuzzi et al., 2014). However, the conidial surface has also been shown to possess proteolytic activity (Asif et al., 2006). A proteomic study on the conidial surface found PEP2, an endopeptidase, and MepB, a metallopeptidase (Asif et al., 2006). Although no evaluation of the effect of *mepB* knockout on conidial interaction with epithelial cells was performed, a previous study found that an *A. fumigatus mepB* mutant strain had no detectable growth phenotype and resulted in a mortality rate comparable to wild type after *in vivo* infection of mice (Jaton-Ogay et al., 1994; Ibrahim-Granet and D’Enfert, 1997). Combined with the lack of significant loss of virulence in a mutant with significantly reduced proteolytic activity (Bergmann et al., 2009), these data suggest that secreted proteases, while likely able to cause damage, may contribute little to the overall virulence of the fungus. A putative extracellular lipase associated with the conidial surface has also been identified in the conidial proteome (Asif et al., 2006). Although little is known about the role of extracellular lipase activity in the pathogenesis of *A. fumigatus*, it has been detected in previous studies (Birch et al., 1996). Further examination of extracellular enzymes, particularly those associated with conidia, is needed to fully understand their importance in the interaction with epithelia and in pathogenesis.

### Gene Expression during Germination and Early Fungal Growth

Aspergillosis is caused primarily by the growth of *A. fumigatus* within the host; therefore, the gene expression patterns that characterize the transition from conidia to germling and to early hyphal growth are important in its pathogenesis. The growth signature of early development is characterized by a rapid increase in protein synthesis. To provide adequate energy for this increase, the metabolism of conidia switches from a low energy, fermentative metabolism to an oxidative metabolism (Lamarre et al., 2008; Teutschbein et al., 2010). Interestingly, transcripts for proteins involved in the catabolism of multiple different carbon sources were found in conidia independent of the growth medium on which the formation of conidia, or conidiation, occurred (Lamarre et al., 2008). This allows for far more rapid germination in a wide variety of carbon sources without requiring a shift in metabolism, and thus a far more adaptive response. For example, resting conidia showed significant alcohol dehydrogenase and pyruvate decarboxylase activity, two key enzymes in alcoholic fermentation (Teutschbein et al., 2010). Nitrogen assimilation is also essential for the rapid growth and germination of *A. fumigatus*, particularly in the relatively nitrogen-poor environment of the lung. *A. fumigatus* germination is regulated by a nitrogen sensing protein kinase (Xue et al., 2004). The rheb small monomeric GTPase gene *rhbA* appears to also have a key role in nitrogen uptake. An increase of *rhbA* expression in early germination has been noted in nitrogen limiting media (Panepinto et al., 2002), as well as in the lungs of mice during early infection with *A. fumigatus* (Zhang et al., 2005).

There are a number of proteins highly abundant in conidia that have some demonstrably important roles in the growth, survival and pathogenesis of conidia. One example is CatA, a conidial catalase that is one of the most abundant conidial proteins and has been shown to protect conidia from oxidative stress, particularly H_2_O_2_ exposure (Paris et al., 2003). Despite this, a mutant lacking the conidial catalase demonstrated no decreased virulence in a mouse model, nor was there any observed increase in killing by alveolar macrophages (Paris et al., 2003). Another protein highly expressed in conidia is the cytoplasmic Cu/Zn superoxide dismutase, Sod1. Loss of Sod1 increased conidial susceptibility to intracellular superoxide ions (Lambou et al., 2010). A triple mutant for *sod1/sod2/sod3* was created in order to investigate the impact of the *sod* genes on virulence (*sod2* and *sod3* encode mitochondrial and cytoplasmic MnSod, respectively). Though no significant effect on virulence was observed, the triple mutant did display severely decreased growth at high temperatures, increased killing by immunocompetent macrophages and a delay in conidial germination (Lambou et al., 2010). While it is possible that the use of immunocompromised mice negated any effects caused by loss of the *sod* genes, the results demonstrate that *sod1* and *sod2* likely have only a minor role in protecting *A. fumigatus* from extracellular superoxide (Lambou et al., 2010). In addition to their role in surface structure and attachment of conidia to extracellular matrix proteins, the conidial hydrophobin RodA has been shown to protect conidia from immune recognition (Aimanianda et al., 2009; Bruns et al., 2010; Carrion et al., 2013). Rodlet proteins such as RodA form an inert layer that masks immunogenic molecules on the cell surface such as β-glucan, the polysaccharide recognized by Dectin-1 (discussed above). Loss of this rodlet layer drastically changes the morphology of the conidial surface, exposing these highly immunogenic elements resulting in a severe impact on conidial survival; conidia lacking a rodlet layer were recognized and killed far more efficiently by neutrophils and other innate immune cells (Thau et al., 1994; Aimanianda et al., 2009; Bruns et al., 2010). Further investigation into these abundant conidial proteins and their impact upon interaction of conidia with epithelial cells could provide new insights into the development of *A. fumigatus* pathogenesis.

To date, only one study has simultaneously examined differential gene expression of both conidia and AECs upon co-incubation. This study identified several gene ontologies in fungi that were up-regulated in conidia as a consequence of their interaction with the respiratory epithelium, such as iron metabolism. Fungal genes related to vacuolar acidification were also up-regulated (Oosthuizen et al., 2011). The reason for this up-regulation is not known; however, V-ATPases are important in other fungi and are implicated in numerous functions including resistance to oxidative stress (Kane, 2007). Formate dehydrogenase, involved in the catabolism of compounds such as methanol, was also up-regulated in conidia upon interaction with AECs (Oosthuizen et al., 2011) as well as in response to neutrophils (Sugui et al., 2008).

## Limitations of Current Cell and Animal Models of Aspergillosis

A good model is essential to obtain relevant data but the choice of model also affects the type of data that can be collected. Cell culture models allow for an examination of the interaction on a cell type to cell type basis; however, the conditions are not necessarily similar to those found *in vivo*. In addition, *in vitro* studies often make use of a single type of cell line cultured in a monolayer, and immortalized lines are often used in order to save time and cost. Although a powerful tool, immortalized cell lines, or indeed even primary cell lines grown in a monolayer, have fundamental differences to cells *in vivo*. Immortalized cell lines do have advantages, such as the ability to propagate indefinitely and respond consistently across years, but they are transformed and therefore may have different responses to stimuli. A good example of this is the A549 alveolar type II cell line. While it does retain a number of the characteristics of alveolar cells *in vivo*, it is a hypotriploid cell line isolated from a carcinoma (Lieber et al., 1976). Results obtained from studies with secondary cell lines should be validated with primary cell lines to confirm results. The majority of studies performed to date have used bronchial, alveolar type II, tracheal, or nasal epithelial cells; as far as the authors of this review are aware, no studies have yet taken place using type I alveolar epithelial cells. To more closely mimic the *in vivo* environment, some researchers have grown cells at an air–liquid interface that results in a pseudostratified epithelium containing differentiated cells organized in a fashion similar to that of the bronchial epithelium (Gruenert et al., 1995). Such models have been used in several studies of *A. fumigatus* lung cell interactions (Botterel et al., 2002, 2008; Khoufache et al., 2010).

While studies performed *in vivo* in animal models most closely imitate the overall host–pathogen interaction, it is much more difficult to collect data specific to a single cell type within the model. However, as techniques for imaging and collecting data on gene expression *in vivo* improve, our ability to examine the specific interactions *in vivo* is increased. There have been several excellent reviews published on animal models of invasive aspergillosis (Clemons and Stevens, 2005, 2006; Patterson, 2005; Paulussen et al., 2014). The inherent differences between human physiology and that of the animal introduce challenges and limitations in the extrapolation of results from animal models. In addition, the expense and ethical considerations limit their use. Nevertheless, *in vivo* animal models provide invaluable information on the importance of various fungal gene products in pathogenesis.

It has been shown that wild type strains of *A. fumigatus* are easily grown in culture and demonstrate little genetic variation; therefore, the results from one strain of fungus are likely to extend to others (Rydholm et al., 2006). The strains used tend to be clinical isolates but no significant genetic clustering of clinical vs. environmental samples has been detected (Debeaupuis et al., 1997; Pena et al., 2015). Nevertheless, the conditions used *in vitro* may not have entirely mimicked those of the lung, resulting in a growth pattern different from that associated with pathogenesis.

## Future Directions

The advent of numerous high-throughput biology techniques in recent years has allowed researchers to collect data on the overall response of both host and pathogen (Culibrk et al., 2016). Although there have been multiple ‘omics’ type studies examining either AECs or *A. fumigatus*, few have examined both simultaneously. Computational modeling of such dual organism expression patterns could indicate potential targets of interest that mediate interactions between the host and pathogen. Such a network has been constructed in a study of the interaction between mice and *Candida albicans* but has yet to be applied to *A. fumigatus* (Tierney et al., 2012).

Recent research has shown that the respiratory epithelium is a key player in an effective immune response to several types of respiratory pathogens. Not only are AECs involved in the recruitment of immune cells, but also they have been shown to participate directly in the elimination of pathogens primarily through the secretion of anti-microbial peptides. Most studies have used transformed cell lines or AECs isolated from ‘normal’ patients. However, AECs from patients at risk of aspergillosis may possess aberrant or insufficient immune responses to conidia and early infection. Hence, studying the epithelium–conidia interaction using cells isolated from susceptible patients could yield novel insights into the role of the respiratory epithelium in host defense against fungal pathogens.

At the time of this writing approximately 95% of ORFs in the *A. fumigatus* genome remain unverified (Cerqueira et al., 2013). The functional characterization of these gene products is highly relevant to the early interaction of AECs with conidia, particularly because conidia contain large numbers of small proteins of unknown or hypothetical function (Suh et al., 2012). Moreover, the disruption of normal eukaryotic cell function by secondary metabolites is of interest, not only due to the ability of secondary metabolites to promote disease progression, but also their potential use as tools to combat other diseases (Li et al., 2013).

The interaction of conidia with cells of the respiratory epithelium is important to our understanding of aspergillosis and the establishment of disease. Elucidation of the specific mechanisms as well as the global responses of both the conidia and the AECs to one another is essential for an understanding of the dynamic interplay between the host and pathogen. An improved understanding of this interaction could lead to more effective treatment and better patient outcomes.

## Author Contributons

CC, LC, MM, and ST all contributed to the content and editing of the manuscript. In addition all authors approved the final manuscript for submission.

## Conflict of Interest Statement

The authors declare that the research was conducted in the absence of any commercial or financial relationships that could be construed as a potential conflict of interest.
